# Perceptual video quality assessment in H.264 video coding standard using objective modeling

**DOI:** 10.1186/2193-1801-3-174

**Published:** 2014-04-04

**Authors:** Ramasamy Karthikeyan, Gopalakrishnan Sainarayanan, Subramaniam Nachimuthu Deepa

**Affiliations:** Teclever Solutions Pvt Ltd, Bangalore, India; HCL Technologies Ltd, Chennai, India; Department of EEE, Anna University, Regional Centre, Coimbatore, India

**Keywords:** H.264, AVC, Perceptual video quality, Mean opinion score [MOS], Blockiness, Jerkiness, Blur

## Abstract

Since usage of digital video is wide spread nowadays, quality considerations have become essential, and industry demand for video quality measurement is rising. This proposal provides a method of perceptual quality assessment in H.264 standard encoder using objective modeling. For this purpose, quality impairments are calculated and a model is developed to compute the perceptual video quality metric based on no reference method. Because of the shuttle difference between the original video and the encoded video the quality of the encoded picture gets degraded, this quality difference is introduced by the encoding process like Intra and Inter prediction. The proposed model takes into account of the artifacts introduced by these spatial and temporal activities in the hybrid block based coding methods and an objective modeling of these artifacts into subjective quality estimation is proposed. The proposed model calculates the objective quality metric using subjective impairments; blockiness, blur and jerkiness compared to the existing bitrate only calculation defined in the ITU G 1070 model. The accuracy of the proposed perceptual video quality metrics is compared against popular full reference objective methods as defined by VQEG.

## Introduction

Digital video in the form of various video applications such as digital television, internet streaming, digital cinema, video on demand, video telephony and video conferencing predominantly engages our life. And these video and multimedia applications are growing fast. In this huge digital video application space, there are various service providers offering solutions to end customers. And the digital video typically goes through different stages of processing before it reaches to the end user, resulted in video quality degradation. So the challenge for these service providers is to guarantee an appropriate Quality of Experience (QoE) for the end user to avoid churn out. Quality assessment for speech has quite a long history and well established, there are extensive work going on to extend the quality assessment to audio and video. The need for an accurate and reliable method of video quality measurement has become more necessary with the new digital video applications and services like mobile TV, streaming video and IPTV. In general, quality measurement has a wide range of uses, such as codec evaluation, headend quality assurance, in-service network monitoring and end equipment quality measurement.

Quality assessment methods can be divided into objective and subjective measurement. Objective methodology uses mathematical models to depict the behavior of the human visual system. Subjective assessment of video quality presents a methodology for video quality assessment that was received by observers and gives opinion about the video that they are viewing. The sum of their opinion gives the Mean Opinion Score (MOS), this provides the measure of subjective quality assessment.

Conscious quality monitoring in an in service mode is beneficial for the service providers and end users. In service quality monitoring techniques required to be low computational complexity, high correlation with MOS and the ability to use the metric meaningfully. Perceptual quality metrics are algorithms designed to model the quality of video and predict end user opinion objectively.

Based on the method of objective metric calculation, they are generally classified as follows (Winkler 2009):

- Data Metrics which measure the fidelity of the signal without considering the content characteristics like Peak signal to noise ratio (PSNR) and mean square error (MSE).
- Picture Metrics which process the visual information in the video data and account for the distortions in the content on perceived video quality.
- Bitstream or Packet parameter based Metric for compressed video delivered over packet network.
- Hybrid metric which is derived based on the combination of above.

Additionally based on the amount of reference information required, they are classified as follows:

- Full Reference (FR) metrics measures the degradation in the test video with respect to a reference video.
- No Reference (NR) metric analyze the test video without the need for an explicit reference clip.
- Reduced Reference (RR) metric is a tradeoff between the FR and NR metric calculation in terms of reference information requirement. The comparison between the test video and the reference video will be based on the extracted information.

Since video compression schemes required to address impairments related to block based prediction on spatial and temporal domain, any calculated metric suited for in service application should be calculated based on picture metrics and no reference model. Because of the advantage of capturing the cumulative effect of the compression on video quality, the picture metric based video quality measurement is proposed in this paper and applied to H.264 compression (ITU-T 2005) scheme for headend quality assurance. Even though the reference video is available in the H.264 encoder, only no reference model is proposed. Since the ability of this proposed scheme should be extended to different in service quality monitoring application and also have low computational complexity.

Because the compression standard is block based and the problem can be generalized over the transform block size, the proposed metric calculation of blockiness, blur and the jerkiness are arrived at block level. This can be generalized to any block based coding and for different size of the transform. We have presented MOS calculation based on impairments of blockiness, blur and jerkiness where the MOS calculation model which carries the cumulative effect of all the three metric. The computation of these impairment metrics are in accordance with the ITU-T P.910 ( 1999) standard. The correctness and effectiveness of these models experimental results are compared against the full reference well known quality assessment method SSIM.

The paper is organized as follows. Section II provides details about the related work in the proposed research area. Section III explains the motivation and proposed perceptual video quality model. Section IV outlines the design and Section V brief about the performance evaluation and discussion. Section VI contains the concluding remarks.

## Related work

Among the different quality metrics used to assess the video quality, an objective full reference quality metric is proposed in Abharana et al. ( 2009) using natural decrease in entropy of decoded frame due to compression and vertical and horizontal artifacts due the blockiness effect and apart from that the spatial and temporal masking properties of human visual system are compared against other standard full reference metrics. But no reference quality metric has more advantage in terms of the computational complexity and the reference availability. Even though there are many works (Brandao et al. 2009; Arum et al. 2012) experimented for quality assessment on the compressed video, there are full reference metric as in Eden ( 2007), proposed a measure of picture quality as peak signal to noise ratio (PSNR) which is a full reference metric and estimated statistically using transform coefficients as no reference metric. A revised PSNR no-reference model is presented in Brandao and Queluz ( 2010) that estimate video quality using estimated DCT coefficients which are derived using Maximum Likelihood techniques. Content spatial-temporal activity calculation based on average SAD and display format based perceptual MOS calculation model is proposed in Joskowicz and Ardao ( 2010) and the relationship between the bit-rate and the MOS is derived. But only using the bit-rate is limiting the estimation quality of certain video service. In Valenzise et al. ( 2012), proposed an estimation of the pattern of lost macroblocks which produces an accurate estimate of the mean-square-error (MSE) distortion introduced by channel errors. The results of the proposed method are well correlated with the MSE distortion computed in full-reference mode, with a linear correlation coefficient of 0.9 at frame level. A two part no reference quality metric calculation consists of training and test is proposed in Kawano et al. ( 2010). In the training phase, they calculate the sensitivity from features like blockiness, blur and edge business etc. and rank these features using the Principal Component Analysis (PCA) method. In Rossholm and Lovstroem ( 2008), the author try to find a linear relationship between quality measurement method and media-layer metrics such as quantization parameter, bits per frame, frame rate, and mean motion vector length. The proposed methods in Ries et al. ( 2007) uses the video quality calculation using parameters such as bit rate, zero length motion vectors, mean motion vector lengths and motion vector direction. Even though bit rate is a key parameter (ITU-T G.1070 2012) for estimating the coding distortion, the subjective quality of different video sequences cannot be correlated well with only the bitrate. So this proposed method, uses impairments such as blockiness, blur and jerkiness introduced by the spatial and temporal activities to improve the estimation accuracy in the encoder for the head end quality assurance.

## Proposed perceptual quality estimation model

The lossy nature of all block based video codecs, compression introduces video artifacts which are noticeable to human visual system. In any application user viewing experience, the video quality is an important factor for the Quality of Experience (QoE). In order to have the QoE defined, quality measurement standardization bodies are trying to define the MOS as measure and define a method of MOS prediction which is reliable and reproducible. Even though some of the objectives are achieved in the existing standards this is being researched to address specific application. The proposed idea in this paper is to arrive at a NR metric based perceptual quality assessment which can be used for continuous monitoring in different applications. At headend this can be implemented as part of the encoder without much complication for the in service assessment of quality of delivery.

Performances of quality assessment methods based on references are limited by the quality of the source video and the video sequence alignment. No reference (NR) based approach is an absolute quality assessment as viewed by the user which is more useful in end to end performance monitoring scenario. Quality assessment is a challenging task when there is no reference. NR method provides advantage of in service real time assessment because of its low computational complexity.

The NR metrics for video blockiness, blur and jerkiness are calculated and the perceptual quality assessment model for the codec for a bitrate is derived in accordance with ITU G.1070. For a set of training and test video sequences, the perceptual quality calculation based on the proposed assessment model is computed and presented. The correctness and effectiveness of this model is experimented and compared against a well known full reference quality metric SSIM as per the methods provided in VQEG.

Video quality parameter I_coding_ for an optimal frame rate is defined in ITU-T G.1070 as follows.1

Where Br_v_ is the bitrate and I_coding_ is coding quality artifacts assessment the value of which will vary from 0 to 4. The perceptual quality metric only for the coding based quality impairments and provides the quality metric at headend.2

And v3, v4 and v5 are constants and any change in v4 impacts the value of MOS greatly, obtaining the value of v4 based on the no reference blockiness, blur and jerkiness is considered. Proposed MOS calculations uses v4 which is a combined scaled distortion indicator as a effect of all the three impairments along with the bitrate.

## Design overview

This section presents the details of the intra frame metrics blockiness and blur and inter frame metric jerkiness at block level. Based on these calculated intra frame and inter frame metrics, the perceptual quality estimation is proposed. The proposed model uses the no reference metrics which also provides reduced computation complexity.

### Blockiness metric

The blockiness metric is measure of the visible edges on the coded picture block boundary; it is calculated based on the Boundary Strength (BS) of the transform block boundaries which is part of the encoder standard. The amount of blockiness present over a widow of frames is accumulated and a normalized blockiness metric (BM) is computed based on this amount of blockiness. BS value of 4 is high blockiness and BS value of 0 is less blockiness. For the calculation of amount of blockiness, all the block boundaries which have BS equal to 4 for intra coded frames and BS equal to 2 for the inter coded frames are counted. This count is accumulated over a frame and based on this the normalized BM metric is calculated and converted to percentage terms. So the value of the BM is between 0 to 100.

### Blur metric

The BLur metric(BL) is defined as loss of energy and spatial details reduction on the sharp edges, if a sharp edge has more depth in the edge pixels, then the image is considered more blurred. This metric is computed using a "Sobel" filter for identifying the sharp edges to calculate the localized blur metric in a frame. Once the blur regions are identified; and then transform coefficients of blocks which fall on the blur region are used for the calculation of blur. Based on the weighted count of each frequency component across all the blocks under computation which is having sufficient number of occurrence compared with low frequency components is computed for the blur count and then this value is normalized to obtain BL. The value of BL will be between 0 to 100.

### Jerkiness metric

Slow camera movement or zoomed video sequences are exposed to jerkiness artifact. This metric is calculated as normalized number of transition between states at macroblock level. Based on a threshold in mean square error, this is been calculated that the macroblock got updated or not. The measure of the status of macro block updation across a window of frames provides the jerkiness artifacts (JR). This is computed as the maximum over time f the standard deviation over space of all the frames. More motion in adjacent frames will result in more value for JR.

**Figure 1:**
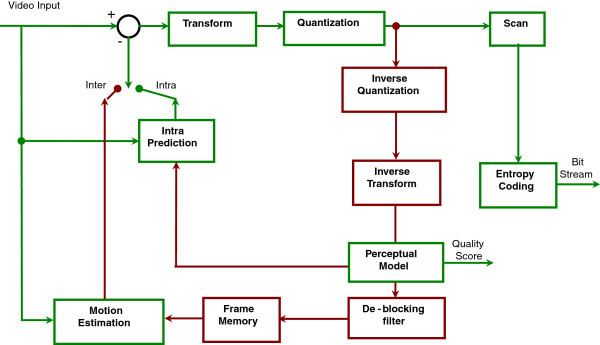
**Block diagram of video coding layer of H.264 encoder with perceptual quality score.**

All the above artifacts are computed as part of H.264 encoder along with the perceptual quality metric calculation as mentioned in the proposed model. And the Figure 1 contemplates a modified block diagram of H.264 encoder where the perceptual quality metric is calculated in service. This provides MOS score for the video sequence along with PSNR, so user can understand the subjective quality of the encoded video.

In the proposed perceptual quality model, the constant v4 is calculated as linear combination of the impairments together. So v4 which is the combined scaled distortion indicator is expressed as follows3

In equation (3), a, b and c are weighted coefficient. These are used to adjust the impact of individual impairment in the perceptual calculation. These values are derived by experiments using the training set of videos and the results are analyzed for set of video content with different spatial and temporal activities. The expected result of each metrics is computed as per the standard P.910 and the training set results with different coefficients are experimented for minimum error.

The computed MOS value as in equation (2) provides the measure of subjective quality of the video sequence. Because the MOS value has the effect of the video impairments blockiness, blur and jerkiness, the testing results shows that this proposed model has high correlation with the standard full reference quality metric SSIM.

The comparison of the accuracy is based on Pearson Correlation Coefficient (PCC) and Root Mean Square Error (RMSE) as proposed in VQEG ( 2003) standard.

## Performance evaluation and discussions

The proposed quality metric calculation is implemented in C language. We have used JM coder for the H.264 video encoding. The metric calculation is implemented as part of JM reference software. The video resolution is of standard definition size and encoding is set to three different bitrates of 512 kbps, 1 mbps and 2 mbps to depict the effect of these impairments at encoder. Four different standard definitions test videos are used to train the weighted coefficients and obtained the constants in Equation (1) and (3) at different bit rate. The training video sequences are "mobile and calendar", "parkrun", "shields" and "stockholm" ( Training video sequence. http://media.xiph.org/). These training test vectors have various spatial and temporal complexities in nature. Since the constant v4 is only the variable one and all others are constant, the perceived quality change will be proportional to the v4 change. Different six video sequences are taken for test purpose; since the parameters are trained there is no need for parameter change for different kind of videos.

**Figure 2:**
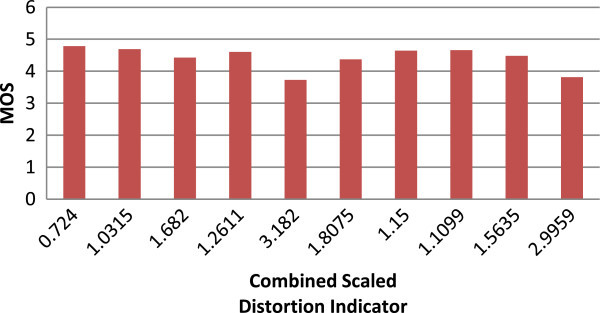
**MOS Vs combined scaled distortion indicator graph for 512 kbps.**

**Figure 3:**
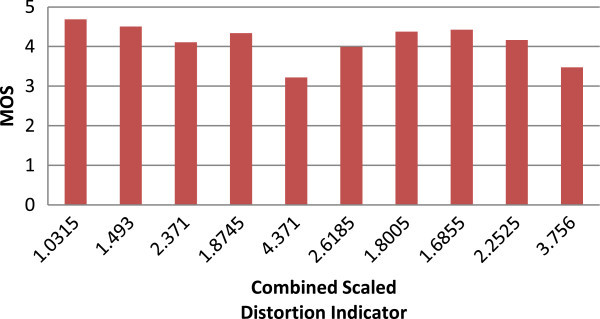
**MOS Vs combined scaled distortion indicator graph for 1 mbps.**

**Figure 4:**
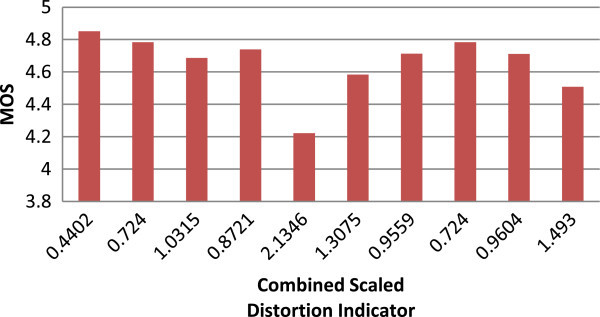
**MOS Vs combined scaled distortion indicator graph for 2 mbps.**

The computations of MOS for these different video sequences are conducted as per the proposed method. Figures 2, 3 and 4 explains the quality metric performance for 512 kbps, 1 mbps and 2 mbps encoding respectively.

The combined scaled distortion indicator value is varying from 1.03 to 4.37 for different test vectors at 512 kbps. And the value of 4.37 for 512 kbps is high compare to 1 mbps which is 3.18, the most distorted video sequence where the test vector has high temporal and spatial complexity. The value for the same is 2.13 for the 2 mbps encoding. The results shows that for different spatial and temporal activities the coding distortion is different apart from that for different bitrate the quality distortion indicator correlates well and these results are compared against SSIM full reference quality metrics based on PCC and RMSE as proposed in VQEG.

The average values of these shows that the proposed model has high correlation for the quality calculation than the well known full reference model SSIM (Wang et al. 2004) and shown in Table 1. The PCC value is high and the RMSE value is less compared to the SSIM model. This shows that the MOS calculated based on the video impairments are more correlated to user viewing experience than standard full reference methods.

**Table 1:** **Performance comparison of proposed model with SSIM**

Video quality assessment model	PCC	RMSE
Proposed model	0.961	0.312
SSIM model	0.763	0.571

This proposed model explains the video artifacts measurements in H.264/AVC coded video related to intra and inter compression which clearly shows the correlation of the calculation is more based on the video impairments method than reference models presented in VQEG. Since the proposed method uses the impairments in the video and a NR method, when using in the decoder end this can capture the combined effect of the encoder, channel. This method can use application which cannot get the full reference or reduced reference information such as broadcasting, IPTV and video telephone etc. Since the parameter training needs to be done for different codec separately. The work can be extended to compare the computation complexity and also to map these impairments parameter from different channel and bitstream information.

## Conclusions

A combined measure of perceived video quality for the H.264/AVC compression is proposed using no reference model. Metrics were implemented in a C/C++ environment as part of JM software of H.264. The objective modeling of subjective quality parameters was derived from the defined standard model. The results are analyzed for correctness with the actual content quality for a given encoding scenario which shows that the values are highly correlated to the users viewing experience. Also these results are compared against a standard full reference model and verified using comparison methods as mentioned in VQEG for a set of training and test vectors. Based on these results, video impairment analysis based quality model which is relatively low computational requirements compared to full reference method was providing better quality indication is evident.
